# Rigid Rest in Rheumatic Carditis

**Published:** 1926

**Authors:** J. A. Nixon

**Affiliations:** Professor of Medicine in the University of Bristol; Physician to the Bristol Royal Infirmary; Consulting Physician to Southmead Hospital


### RIGID REST IN RHEUMATIC CARDITIS.

BY

J. A. NIXON, C.M.G., M.D. Cantab., F.R.C.P.,

*Professor of Medicine in the University of Bristol;*
*Physician to the Bristol Royal Infirmary;*
*Consulting Physician to Southmead Hospital.*

---

RHEUMATISM in childhood has been calculated by Dr. Gordon Pugh[1] in a report to the Metropolitan Asylums Board as being "eventually responsible for one-twenty-fourth of all deaths."

It is a good many years since (in 1910) I first tried the effect of prolonged rest in bed for a child in a first attack of rheumatic fever. There was evidence of carditis, for which he stayed in bed for close on a year and improved remarkably. It seemed to me that a heart whose apex beat was for many months displaced outwards, and whose action was unduly rapid, rather suddenly became, after many months, markedly quieter in its action and smaller. There was a systolic bruit that became less audible.

Every writer since Francis Sibson[2] has advocated rest for the rheumatic heart. Sibson seems to have been the first to insist upon a "rigid system of rest." The connection between rheumatic fever and heart disease had been recognised before his time, but he brought figures to show how valuable rest in rheumatic fever could be in lessening the liability to heart complications. Sibson was a physician at St. Mary's Hospital, and was largely responsible for the interest which Mary's men have taken in cardiology since his day. "The influence of the treatment of acute rheumatism by means of rest," he wrote, "on the occurrence, severity and permanent ill-effects of endocarditis will be best illustrated by comparing the clinical history of the 94 cases treated by rest with that of the 325 cases not so treated."

There was endocarditis alone, or combined with pericarditis, in one-half (161 in 325) of the first series of cases that were not treated upon a system of absolute rest; and in two-fifths (34 in 94) of the series that were so treated.

"Thus far," as Sibson says, "the comparison is but slightly in favour of the treatment of acute rheumatism by a rigid system of rest." He goes on to compare the number of cases previously unaffected with endocarditis which acquired permanent valvular disease so as to injure health during the remainder of life and to shorten life itself, also the number of cases which escaped all evidence of endocarditis, and concludes that "the whole chain of evidence points irresistibly to the conclusion that the extent, severity and permanent ill-effects of the endocarditis were much greater in the series of cases that were not rigidly treated by rest than in the series that were so treated." Of pericarditis he says more than twice as many of the series of cases that were not treated by rest were attacked with pericarditis than of the series of cases that were treated by a rigid system of rest.

Now we are all agreed upon a point that was not so generally admitted in 1877 when Sibson wrote for *Reynolds' System of Medicine.* At the discussion on "Rheumatic Infection in Children" at the British Medical Association's meeting in Bath[3] last year there seemed a consensus of opinion that prolonged rest was necessary for children suffering from rheumatic carditis; there was, however, no definite idea as to how long that rest should continue. Dr. Askins urged the desirability of establishing open-air country hospital schools, where cases can have proper rest, education and recreation.

Sir James Mackenzie[4] had laid down the rule that we should "keep the patient strictly in bed until there is assurance that the condition is quite abated . . . until the rate is quite normal, even though six months may elapse before this result is brought about."

Sutherland[5] went further: he advocated "prolonged rest in bed, six months or longer if necessary . . . and sanatorium treatment for one or two years."

Sir Archibald Garrod[6] has expressed his confident belief that if it were possible to give two or three months' rest in bed to all children with rheumatic fever, heart disease amongst adults would be very materially reduced, both as to severity and frequency.

Dr. Phillips, Medical Superintendent of Southmead Hospital, and I have had the opportunity of seeing the results of prolonged rest in bed of several children suffering from rheumatic fever and chorea with early carditis.

It is almost impossible at present to lay down any standard by which to compare results of cases treated by means of Sibson's "rigid system of rest" with those not so treated. Sibson found that cases of rheumatic fever were less often attacked by carditis when kept rigidly at rest. We may accept this as proved beyond a shadow of doubt.

What happens to patients, treated by a rigid system of rest, whose hearts are affected, as they will be in some proportion of cases?

My own impression has been that these patients escape with less serious damage to the heart. We have brought some here to-night for you to judge. I must admit that these patients do not encourage us to hope that a sound heart will result from prolonged rest in bed even when the child has been in hospital before the very earliest sign of cardiac involvement was observed. One of these children, a girl of thirteen, came into the Royal Infirmary with acute rheumatism, and there developed endo- and pericarditis in a heart that previously appeared sound. She remained in bed continuously for fifteen months at the Infirmary and subsequently at Southmead. But you can judge for yourselves of the permanent damage that remains ; there is the loud systolic bruit at the apex, the large heart, and the poor response to effort. I am afraid she is destined to be a life-long "cardiac cripple." This is typical of our experience: sometimes we congratulate ourselves on the surprising ultimate recovery, and sometimes we are bound to confess that the results are no better than if the child had been sent home to run the streets.

I have carefully considered the points to be observed in deciding whether a child should still be kept in bed. These points seem to me to fall into two groups :—

*General.*—Cessation of pyrexia. Abatement of joint pains and choreic movement. Relief of cyanosis or dyspnœa.

*Local Cardiac.*—Rate and rhythm of heart. Position of apex beat. Size of cardiac dulness. Disappearance of bruit. Clearing up of pericardial signs. Size of heart by X-ray. Electro-cardiograph.

But, however carefully we attend to these points, either separately or collectively, we seem to have little or no control over the result.

Is Garrod's view correct that by keeping rheumatic children long enough in bed heart disease in adults could be prevented ?

Dr. Gordon Pugh in a second report[7] speaks of the experience gathered at St. Mary's Home at Broadstairs by Dr. Martin Raven:

" During three and a half years ninety-two children were treated for rheumatic infection, with an average stay of six months. Last summer forty-six of those who had been discharged were re-examined, and it was found that eight patients (17 per cent.) had developed fresh acute rheumatism shortly after returning home. The numbers are too small to allow of accurate deduction, but the result, so far as it goes, does not encourage the view that prolonged stay in a special institution will prevent relapses, and one would hesitate to advise the provision of accommodation on a large scale for that specific purpose."

One begins to wonder whether rheumatic fever, like syphilis and encephalitis lethargica, is a disease which throws out no recognisable signal of arrest, and has no known criterion of cure. We must all have seen the length of time during which choreic movements may persist in Sydenham's chorea without pyrexia or cardiac lesion. Presently an acute pyrexial exacerbation occurs and the heart becomes affected. The disease in such a case would, no doubt, have been regarded as cured if the movements caused by a chance involvement of the *corpus striatum* had not continued.

There must be far more cases in which the disease lies absolutely quiescent (because that delicate recording instrument the *corpus striatum* remains untouched), and yet the infection is not become avirulent.

Is a new attack of rheumatic fever merely a relapse in the course of a single infection? When, if ever, does the infection of rheumatic fever lose its virulence?

These considerations make us pause in our advocacy of sanatorium treatment for rheumatic children. Will they ever be more than homes for cripples? Will they prevent the crippledom?

The experience that Dr. Phillips and I have had of trying to stem the progress of rheumatic carditis by a "rigid system of rest" does not support Garrod's view that by such measures heart disease in adults can be prevented. We must reflect, too, that not every heart becomes desperately damaged even if the rest treatment is quite inadequate. I am inclined to repeat the warning of Dr. J. A. Glover,[3] that it is rheumatic fever we must study to prevent, and that we really know very little about the etiology of the disease. But we know that rheumatic fever can be, and habitually is, prevented from attacking the children of a certain social grade, because rheumatic fever is practically non-existent in our great Public Schools.

There is obviously a social factor at work which ought not to be undiscoverable, even if the causal organism eludes us. Instead of accepting rheumatic fever as inevitable and trying to cure its cardiac victims by prolonged rest, I prefer to advocate the prevention of rheumatic fever by means of the "social factor," if I knew how to define it except in terms of taxable income.

### DISCUSSION.

DR. ALEXANDER asked whether the disposition of the patient could be taken as any evidence of persistent rheumatic infection after other signs had subsided.

He related one case, observed for ten years, in which, although the patient had risen from bed quite early after the subsidence of carditis, no evil effect had been found. He pleaded especially for the co-ordination of records of these cases as they progressed through life. He felt that if children strongly opposed rest in bed this might be a good criterion of the cessation of need for such treatment.

Dr. J. O. Symes instanced the case of a child who had had chorea without cardiac signs. Should such a child be kept in bed for months ? When we found a case with a definite murmur and subacute rheumatism we must ask ourselves whether this was a new or an old lesion. If new, months of rest would be indicated, but if not it was not easy to see what good this could do. This disease was rare in private practice ; permanent lesions were still more rare. He thought this was probably on account of better home circumstances ; the chances of infection and re-infection were much less. The lower middle class children did not get relapses when in good surroundings, but tended to do so when they returned home, even after a long rest.

Dr. Herapath asked whether the provision of prolonged rest did more than postpone relapses. In all cases there was myocarditis, although there might be no obvious signs. Such cases, he thought, had permanent damage to the heart muscle.

Dr. Carleton said that he had been using the return of the normal sinus arrhythmia as an index of healing of the cardiac lesion.

Dr. Todd agreed as to the value of loss of the sinus response as an indication for the need for continued rest in treatment.

DR. CAREY COOMBS considered that the problem is not a mechanical one ; the body has to deal with a persistent infection. As in tuberculosis, prolonged rest under favourable conditions produces definite improvement. He thought that the infected heart in the same way should be rested. Educational treatment centres are needed ; the subjects of heart disease are unfit for the general labour market. The education of heart patients might be combined with that of poliomyelitis subjects. In America this plan had been found successful.

DR. NIXON, in reply, stated that he did not consider that opposition to treatment was a good index that rest is not needed. Even where there was no evidence of cardiac disease he still thought prolonged rest was often indicated ; in any case of doubt, it was safer to order prolonged rest. Probably nutrition rather than environment was the important factor in preventing attacks and recurrences. He agreed that return of the normal sinus arrhythmia was a valuable sign of present recovery of the heart, but it told nothing of latent infection.

REFERENCES.

[1] W. T. Gordon Pugh, Report on "Rheumatic Infection in Childhood," Metropolitan Asylums Board, December 7th, 1925.

[2] F. Sibson, "Endocarditis," *Reynolds' System of Medicine*, 1877, iv. 526.

[3] *Brit. M. J.*, 1925, ii. 788.

[4] Sir J. Mackenzie, *Principles of Diagnosis and Treatment of Heart Affections*, 2nd Ed., p. 147.

[5] G. A. Sutherland, *Diseases of Children*, 1907, p. 170.

[6] Sir A. E. Garrod, quoted by Pugh, *vide* ref. 1.

[7] W. T. Gordon Pugh, Second Report on "Rheumatic Infection in Childhood," Metropolitan Asylums Board, Feb. 6th, 1926.

